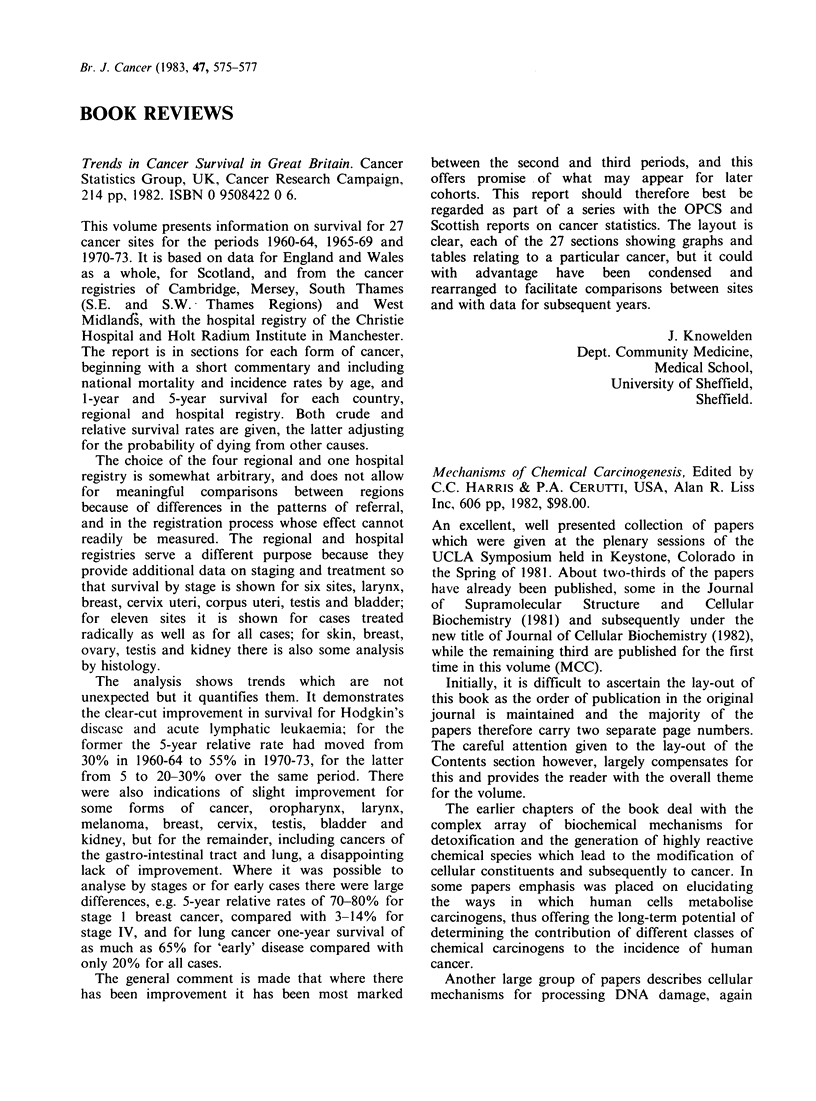# Trends in Cancer Survival in Great Britain

**Published:** 1983-04

**Authors:** J. Knowelden


*Br. J. Cancer* (1983, **47**, 575–577

### BOOK REVIEWS

*Trends in Cancer Survival in Great Britain.* Cancer Statistics Group, UK, Cancer Research Campaign, 214 pp, 1982. ISBN 0 9508422 0 6.

This volume presents information on survival for 27 cancer sites for the periods 1960-64, 1965-69 and 1970-73. It is based on data for England and Wales as a whole, for Scotland, and from the cancer registries of Cambridge, Mersey, South Thames (S.E. and S.W. Thames Regions) and West Midlands, with the hospital registry of the Christie Hospital and Holt Radium Institute in Manchester. The report is in sections for each form of cancer, beginning with a short commentary and including national mortality and incidence rates by age, and 1-year and 5-year survival for each country, regional and hospital registry. Both crude and relative survival rates are given, the latter adjusting for the probability of dying from other causes.

The choice of the four regional and one hospital registry is somewhat arbitrary, and does not allow for meaningful comparisons between regions because of differences in the patterns of referral, and in the registration process whose effect cannot readily be measured. The regional and hospital registries serve a different purpose because they provide additional data on staging and treatment so that survival by stage is shown for six sites, larynx, breast, cervix uteri, corpus uteri, testis and bladder; for eleven sites it is shown for cases treated radically as well as for all cases; for skin, breast, ovary, testis and kidney there is also some analysis by histology.

The analysis shows trends which are not unexpected but it quantifies them. It demonstrates the clear-cut improvement in survival for Hodgkin's disease and acute lymphatic leukaemia; for the former the 5-year relative rate had moved from 30% in 1960-64 to 55% in 1970-73, for the latter from 5 to 20–30% over the same period. There were also indications of slight improvement for some forms of cancer, oropharynx, larynx, melanoma, breast, cervix, testis, bladder and kidney, but for the remainder, including cancers of the gastro-intestinal tract and lung, a disappointing lack of improvement. Where it was possible to analyse by stages or for early cases there were large differences, e.g. 5-year relative rates of 70–80% for stage 1 breast cancer, compared with 3–14% for stage IV, and for lung cancer one-year survival of as much as 65% for 'early' disease compared with only 20% for all cases.

The general comment is made that where there has been improvement it has been most marked between the second and third periods, and this offers promise of what may appear for later cohorts. This report should therefore best be regarded as part of a series with the OPCS and Scottish reports on cancer statistics. The layout is clear, each of the 27 sections showing graphs and tables relating to a particular cancer, but it could with advantage have been condensed and rearranged to facilitate comparisons between sites and with data for subsequent years.

J. Knowelden
Dept. Community Medicine,
Medical School,
University of Sheffield,
Sheffield.